# Beyond detection: quantitative interpretation of *Aspergillus*-positive bronchoalveolar lavage fluid metagenomic next-generation sequencing for diagnostic stratification and prediction of respiratory deterioration

**DOI:** 10.3389/fcimb.2026.1897649

**Published:** 2026-07-21

**Authors:** Yan Wang, Jinghan Lei, Shunyao Cui, Peixi Zhou, Yuanxing Wu

**Affiliations:** 1 Department of Pulmonary and Critical Care Medicine, Beijing Anzhen Hospital, Capital Medical University, Beijing, China; 2 School of Public Health, Capital Medical University, Beijing, China

**Keywords:** antifungal therapy, *Aspergillus*, bronchoalveolar lavage fluid, fungal burden, invasive pulmonary aspergillosis, metagenomic next-generation sequencing, respiratory deterioration

## Abstract

**Background:**

The increasing use of bronchoalveolar lavage fluid (BALF) metagenomic next-generation sequencing (mNGS) has substantially improved the detection of *Aspergillus* species in patients with suspected pulmonary infections. However, positive mNGS results frequently present a clinical dilemma because *Aspergillus* may represent invasive pulmonary aspergillosis (IPA), airway colonization, or transient fungal detection. The clinical value of quantitative fungal burden assessment remains insufficiently defined, particularly regarding risk stratification among untreated patients.

**Methods:**

We conducted a retrospective real-world cohort study including 114 hospitalized patients with BALF mNGS-positive *Aspergillus* detected between April 2024 and November 2025. Patients were classified according to clinical IPA diagnosis, antifungal treatment status, and occurrence of respiratory deterioration during a 3-month follow-up period. Quantitative fungal burden was expressed as reads per ten million (RPTM). Receiver operating characteristic (ROC) analysis, logistic regression, integrated discrimination improvement (IDI), and category-free net reclassification improvement (NRI) were used to evaluate diagnostic and prognostic performance.

**Results:**

Among 114 patients, 31 met clinical diagnostic criteria for IPA and 83 were classified as non-IPA. *Aspergillus* burden was significantly higher in IPA patients than in non-IPA patients (logarithmic scale median RPTM 2.46 vs. 0.30, P < 0.001). ROC analysis identified an exploratory cohort-derived diagnostic threshold of 75 RPTM for IPA discrimination (AUC = 0.853, 95% CI 0.745–0.960). Among 77 patients who did not receive antifungal therapy, 31 experienced respiratory deterioration during follow-up. Higher RPTM values were independently associated with deterioration (adjusted OR = 5.27, 95% CI 1.78–17.06, P = 0.001). An exploratory RPTM threshold of 2.5 showed modest discriminatory ability for subsequent respiratory deterioration, with an AUC of 0.682. Incorporation of quantitative fungal burden significantly improved discrimination and reclassification performance beyond conventional clinical variables. In contrast, baseline RPTM showed no significant association with respiratory deterioration among patients receiving antifungal therapy.

**Conclusions:**

Quantitative interpretation of *Aspergillus*-positive BALF mNGS results may provide additional information beyond simple pathogen detection. Two exploratory cohort-derived thresholds were identified: a higher threshold associated with clinical IPA adjudication and a lower threshold associated with subsequent respiratory deterioration among untreated patients. These findings are hypothesis-generating and require external validation before clinical application. RPTM should be interpreted as an adjunctive marker within the overall clinical context rather than as a standalone diagnostic or prognostic threshold.

## 1 Introduction

Invasive pulmonary aspergillosis (IPA) remains one of the most severe fungal infections affecting patients with impaired host defenses and chronic respiratory diseases (Kousha et al., 2011; Patterson et al., 2016; Donnelly et al., 2020). Although IPA has historically been associated with neutropenia, hematologic malignancies, and other classical immunocompromising conditions, its epidemiological spectrum has expanded to include patients with chronic obstructive pulmonary disease (COPD), bronchiectasis, prolonged corticosteroid exposure, and critically ill patients without traditional host factors; in the intensive care setting, diabetes mellitus has also been reported as a potential risk factor for invasive aspergillosis (Bulpa et al., 2007; Trof et al., 2007; Blot et al., 2012; Taccone et al., 2015; Schauwvlieghe et al., 2018; Tiew et al., 2022; Liu et al., 2025).

Timely diagnosis of IPA remains challenging. Clinical manifestations are nonspecific and radiological findings frequently overlap with bacterial, viral, and noninfectious pulmonary diseases (Blot et al., 2012; Patterson et al., 2016). Conventional microbiological approaches, including fungal culture, galactomannan (GM) assays, and histopathology, are constrained by variable sensitivity across host populations and specimen types, prolonged turnaround time, or invasiveness (Barton, 2013; Zou et al., 2012; Patterson et al., 2016).

Metagenomic next-generation sequencing (mNGS) has emerged as a powerful culture-independent diagnostic modality capable of unbiased pathogen detection directly from respiratory specimens (Chiu and Miller, 2019). Increasing use of BALF mNGS has improved pathogen detection and provided an important complementary approach for recognizing pulmonary aspergillosis, particularly in non-neutropenic and critically ill populations (Bao et al., 2022; Jia et al., 2023; Zhu et al., 2023; Niu et al., 2024). However, improved detection has simultaneously created a new diagnostic challenge: interpretation of positive results (Jiang et al., 2024; Liu et al., 2024).

Unlike many bacterial pathogens, *Aspergillus* species frequently colonize damaged airways without causing invasive disease (Vandewoude et al., 2006; Gago et al., 2019; Tiew et al., 2022). Therefore, a positive mNGS result does not necessarily indicate active infection requiring antifungal therapy. Distinguishing invasive disease from colonization remains particularly difficult in patients with chronic respiratory disorders and non-classical host factors (Blot et al., 2012; Jiang et al., 2024).

Several recent studies have suggested that quantitative sequencing burden may provide clinically relevant information beyond qualitative positivity (Jia et al., 2023; Jiang et al., 2024; Liu et al., 2024). Nevertheless, available evidence remains limited, and no widely accepted quantitative interpretation framework currently exists for *Aspergillus*-positive BALF mNGS results (Jiang et al., 2024; Liu et al., 2024; Niu et al., 2024). Furthermore, little is known regarding whether quantitative fungal burden can predict future clinical deterioration among patients who do not initially receive antifungal therapy.

Accordingly, we conducted a real-world cohort study to evaluate the clinical significance of quantitative *Aspergillus* burden measured by BALF mNGS. We sought to:

Determine whether quantitative fungal burden distinguishes clinically diagnosed IPA from non-IPA patients;

Explore its prognostic value for respiratory deterioration;

Assess its incremental value beyond conventional clinical markers;

Develop a clinically interpretable quantitative framework for *Aspergillus*-positive BALF mNGS results.

## 2 Materials and methods

### 2.1 Study design and participants

We conducted a retrospective single-center cohort study in the Department of Respiratory and Critical Care Medicine, Beijing Anzhen Hospital, Capital Medical University. Consecutive hospitalized adult patients who underwent BALF mNGS between April 2024 and November 2025 and had *Aspergillus* reads detected were screened. Clinical data, laboratory findings, radiological characteristics, microbiological results, treatment decisions, and follow-up outcomes were extracted from the electronic medical record system. The study was approved by the Ethics Committee of Beijing Anzhen Hospital, Capital Medical University (2026169X).

Patients were eligible if they were aged 18 years or older and had BALF mNGS results showing *Aspergillus* reads of at least 1. Exclusion criteria were age younger than 18 years, severe missingness of key data required to determine infection status, treatment, or outcomes, repeat mNGS testing within 14 days of an index test, and active discontinuation of therapy or discharge against medical advice precluding evaluation of treatment response or prognosis. When two samples from the same patient were obtained within 14 days, they were considered one infectious episode and only the first *Aspergillus*-positive BALF mNGS result was included.

### 2.2 Conventional microbiological testing

BALF, blood, sputum, and other respiratory samples were evaluated using conventional microbiological testing when clinically indicated. Bacterial cultures were performed on blood agar, chocolate agar, and MacConkey agar. Fungal cultures were performed on Sabouraud agar. Fungal biomarkers included serum or BALF 1,3-beta-D-glucan and galactomannan testing. Additional tests included *Cryptococcus* antigen, T-SPOT.TB, GeneXpert for *Mycobacterium* tuberculosis, smear microscopy for fungi using fluorescent or potassium hydroxide staining, and acid-fast staining for mycobacteria. All BALF samples underwent bacterial and fungal culture.

### 2.3 mNGS workflow and quantification

BALF samples were submitted to Novogene Medical Laboratory for PD-seq metagenomic DNA sequencing. DNA was extracted from 400 microliters of BALF using the Novogene PD-seq metagenomic DNA extraction kit and libraries were constructed using the Novogene PD-seq metagenomic DNA library preparation kit. DNA fragments of approximately 150 to 200 base pairs were generated and underwent end repair, phosphorylation, A-tailing, adaptor ligation, purification, and PCR amplification. Sequencing was performed on an Illumina platform with single-end reads of 50 or 75 base pairs, generating at least 20 million reads per sample.

Raw sequencing data were filtered using fastp to remove adaptors, low-quality reads, short reads, and low-complexity sequences. Human reads were removed by alignment to the human reference genome using Bowtie2. Remaining reads were aligned to the PD-seq microbial database using BWA. The database included bacteria, viruses, fungi, and parasites. Species abundance, coverage, and sequencing depth were calculated using validated bioinformatics procedures. Negative controls consisting of sterile nucleic acid-free water and noninfected plasma controls were processed simultaneously to monitor contamination.

*Aspergillus* positivity was defined according to the laboratory-validated bioinformatics pipeline. In brief, microbial read counts had to exceed the corresponding negative control by at least threefold. Organisms with high detection rates in negative controls were considered potential environmental contaminants and removed. Suspected positive pathogens were confirmed by BLAST verification when required. Quantitative *Aspergillus* burden was represented by reads per ten million (RPTM), a normalized semi-quantitative index used for subsequent analyses.

### 2.4 Clinical diagnosis of IPA

The clinical diagnosis of IPA was adjudicated with reference to the revised EORTC/MSGERC definitions and the IDSA aspergillosis guideline. According to the revised EORTC/MSGERC framework, IPA cases were further reviewed and categorized as proven, probable, or possible IPA whenever sufficient clinical, radiological, and mycological information was available. Proven IPA required histopathological or direct microscopic evidence of tissue invasion by *Aspergillus*. Probable IPA was defined by the presence of compatible host factors, clinical or radiological features, and mycological evidence. Possible IPA was considered when compatible clinical and radiological findings were present but mycological evidence was insufficient or incomplete. Because histopathological confirmation was not available in most patients and the cohort included many non-neutropenic patients with chronic respiratory diseases or metabolic comorbidities, the final clinically diagnosed IPA group mainly consisted of probable and clinically suspected IPA cases after multidisciplinary adjudication.

Microbiological evidence included BALF or serum galactomannan positivity and isolation of *Aspergillus* from sputum or BALF culture. BALF mNGS detection of *Aspergillus* was considered supportive evidence only in the appropriate clinical context, whereas the quantitative RPTM value was not used for IPA adjudication. To minimize incorporation bias, two experienced clinicians independently classified patients as clinically diagnosed IPA or non-IPA while blinded to *Aspergillus* RPTM values, ROC-derived thresholds, and subsequent statistical results. Disagreements were resolved by discussion with a third senior clinician or by multidisciplinary consensus.

### 2.5 Follow-up and respiratory deterioration

Follow-up information was obtained from inpatient and outpatient electronic medical records. The follow-up period started at discharge or completion of the index hospitalization and continued for 3 months. Clinical symptoms, complications, medication adjustments, radiological changes, unplanned revisits, and rehospitalizations were recorded.

As no standardized definition of respiratory deterioration is available for patients with *Aspergillus*-positive BALF mNGS results, respiratory deterioration was defined *a priori* as a composite clinical outcome reflecting worsening respiratory status or disease progression during follow-up. Respiratory deterioration was considered present when at least one of the following occurred: persistent or worsening respiratory symptoms, including recurrent fever, increased cough or sputum production, worsening dyspnea, chest pain, hemoptysis, or overall deterioration in respiratory status; radiographic progression requiring clinical reassessment, including enlargement or worsening of pre-existing pulmonary lesions or the appearance of new nodules, consolidation, ground-glass opacities, cavitation, or more extensive bilateral involvement; or unplanned rehospitalization for respiratory disease. Events clearly attributable to bacterial infection, viral infection, heart failure, pulmonary embolism, malignancy progression, or non-respiratory causes were not classified as respiratory deterioration when sufficient clinical evidence was available.

### 2.6 Statistical analysis

Statistical analyses were performed using SPSS 26.0, GraphPad Prism 10.1.2, and RStudio with R 4.5.2. Continuous variables were tested for normality using the Shapiro-Wilk test. Normally distributed variables are presented as mean ± SD and compared using the t test. Non-normally distributed variables are presented as median and interquartile range and compared using the Mann-Whitney U test. Categorical variables are presented as number and percentage and compared using chi-square or Fisher exact tests.

ROC curve analysis was used to evaluate the discriminatory performance of RPTM for clinical IPA diagnosis and respiratory deterioration. Exploratory cohort-derived thresholds were determined using the Youden index. Binary logistic regression was used to identify variables associated with respiratory deterioration. Incremental predictive performance was assessed by IDI and category-free NRI. Two-sided P values less than 0.05 were considered statistically significant.

## 3 Results

### 3.1 Patient enrollment and baseline characteristics

A total of 130 *Aspergillus*-positive BALF mNGS samples were screened. After excluding repeat samples obtained within 14 days from the same patient (n = 3) and samples with incomplete clinical data (n = 13), 114 patients were included in the final analysis. Thirty-one patients were classified as clinically diagnosed IPA and 83 as non-IPA. According to the EORTC/MSGERC definitions, no patient met the criteria for proven IPA, while all 31 clinically diagnosed IPA patients fulfilled the criteria for probable IPA. In addition, 2 patients in the non-IPA group met the criteria for possible IPA based on host factors and radiological features but were not classified as clinically diagnosed IPA after multidisciplinary adjudication because of insufficient mycological evidence and/or alternative clinical explanations.

**Figure 1:**
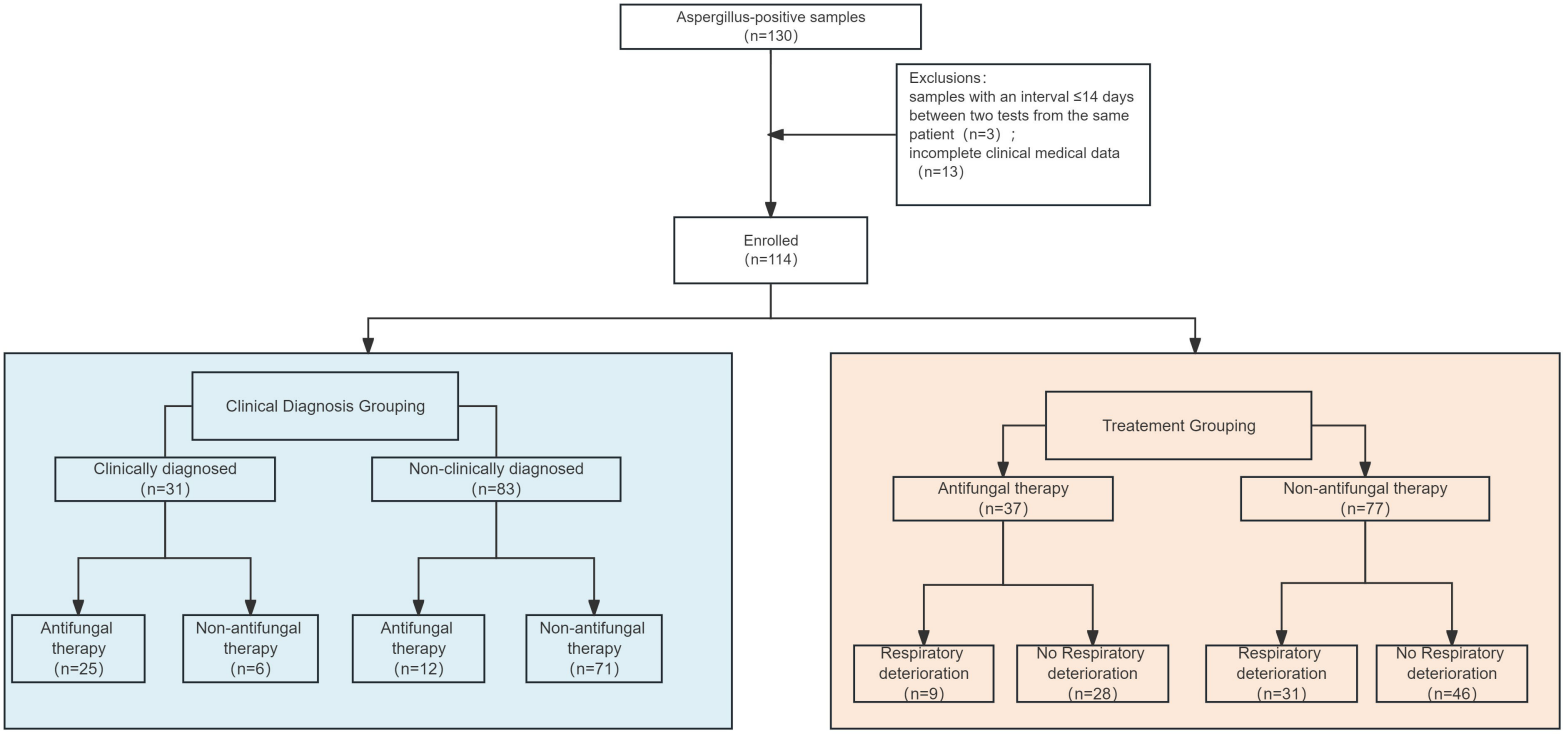
Flowchart of study enrollment and grouping. A total of 130 *Aspergillus*-positive BALF mNGS samples were screened; 16 were excluded, leaving 114 patients for the final analysis. Patients were grouped by clinical diagnosis and antifungal treatment status. Treated indicates patients who received antifungal therapy; untreated indicates patients who did not receive antifungal therapy. The number of patients in each subgroup is shown in the flowchart. Respiratory deterioration during the 3-month follow-up was further assessed in each treatment group.

According to treatment status, 37 patients received antifungal therapy, including 25 clinically diagnosed IPA patients and 12 non-IPA patients. The remaining 77 patients did not receive antifungal therapy, including 6 clinically diagnosed IPA patients and 71 non-IPA patients. During the 3-month follow-up, respiratory deterioration occurred in 9 patients in the antifungal-therapy group and 31 patients in the non-antifungal-therapy group, whereas 28 and 46 patients, respectively, did not experience respiratory deterioration (**Figure 1**).

### 3.2 Baseline characteristics of *Aspergillus*-positive patients

Among the 114 patients included in the analysis, the median age was 66 years (IQR, 56–74 years), and 54 patients were male (47.4%). The median white blood cell, lymphocyte, and neutrophil counts were 6.52 × 10^9/L (IQR, 4.98–9.23 × 10^9/L), 1.28 × 10^9/L (IQR, 0.88–1.68 × 10^9/L), and 4.35 × 10^9/L (IQR, 3.00–6.95 × 10^9/L), respectively. The median mNGS RPTM value was 3.74 (IQR, 1.00–54.67), and serum GM positivity was observed in 21 patients (18.4%).

In terms of clinical manifestations, cough was the most frequently reported symptom, occurring in 90 patients (78.9%), followed by sputum production in 84 patients (73.7%), dyspnea in 62 patients (54.4%), fever in 35 patients (30.7%), and hemoptysis in 9 patients (7.9%).

Regarding chest imaging findings, bilateral lung involvement was observed in 69 patients (60.5%). Multiple nodules were present in 45 patients (39.5%), patchy opacities in 40 patients (35.1%), micronodules in 28 patients (24.6%), ground-glass opacities in 27 patients (23.7%), cavitary lesions in 2 patients (1.8%), and the halo sign in 1 patient (0.9%).

With respect to underlying diseases, diabetes mellitus was present in 65 patients (57.0%). Chronic obstructive pulmonary disease, bronchiectasis, and bronchial asthma were identified in 27 (23.7%), 24 (21.1%), and 14 patients (12.3%), respectively. Overall, 54 patients (47.4%) had an immunosuppressive status (**Table 1**).

**Table 1:** Baseline clinical characteristics, underlying diseases, and imaging findings of patients with *Aspergillus*-positive mNGS results.

Parameters	mNGS positive (n = 114)
Age, median (IQR)	66 (56, 74)
Male, n (%)	54 (47.4)
Clinical laboratory tests, median (IQR)	
WBC (×10^9/L)	6.52 (4.98, 9.23)
PCT (ng/mL)	0.04 (0.02,0.07)
CRP (mg/L)	5.77 (1.52, 36.34)
Lymphocytes (×10^9/L)	1.28 (0.88, 1.68)
Neutrophils (×10^9/L)	4.35 (3.00, 6.95)
D-dimer (ng/mL)	174.50 (103.25, 488.00)
mNGS RPTM	3.74 (1.00, 54.67)
Serum GM positive, n (%)	21 (18.4)
Clinical symptoms, n (%)	
Cough	90 (78.9)
Expectoration	84 (73.7)
Dyspnea	62 (54.4)
Fever	35 (30.7)
Hemoptysis	9 (7.9)
Imaging findings, n (%)	
Bilateral lung involvement	69 (60.5)
Multiple nodules	45 (39.5)
Patchy shadow	40 (35.1)
Micronodule	28 (24.6)
Ground-glass opacity	27 (23.7)
Cavitary lesion	2 (1.8)
Halo sign	1 (0.9)
Underlying diseases, n (%)	
Diabetes mellitus	65 (57.0)
COPD	27 (23.7)
Bronchiectasis	24 (21.1)
Bronchial asthma	14 (12.3)
Immunosuppression, n (%)	54 (47.4)

IQR, interquartile range; BMI, body mass index; WBC, white blood cell; PCT, procalcitonin; CRP, C-reactive protein; mNGS, metagenomic next-generation sequencing; RPTM, reads per ten million; COPD, chronic obstructive pulmonary disease.

Among the 114 *Aspergillus*-positive patients, species-level identification by mNGS revealed that *Aspergillus fumigatus* was the predominant species, accounting for 42 patients (36.8%), followed by *Aspergillus flavus* in 30 patients (26.3%), *Aspergillus niger* in 25 patients (21.9%), *Aspergillus tubingensis* in 5 patients (4.4%), *Aspergillus nidulans* in 3 patients (2.6%), *Aspergillus terreus* in 2 patients (1.8%), and *Aspergillus glaucus* in 2 patients (1.8%). Mixed *Aspergillus* species were observed in 2 patients (1.8%), and other species (*A. ustus, A. lentulus, A. calidoustus*) were detected in 1 patient each (0.9%, respectively). Because the number of patients with each non-fumigatus or potentially cryptic species was small, species-specific RPTM thresholds were not analyzed.

Immunosuppression was defined as the presence of any of the following conditions: primary immunodeficiency disease; active malignancy, excluding localized skin cancer and early-stage malignancies; prolonged corticosteroid therapy, defined as continuous treatment for >14 days; lymphocyte count <0.5 × 10^9/L; or neutrophil count <1.0 × 10^9/L.

### 3.3 Quantitative *Aspergillus* burden differentiates clinically diagnosed IPA from non-clinically diagnosed IPA

**Figure 2:**
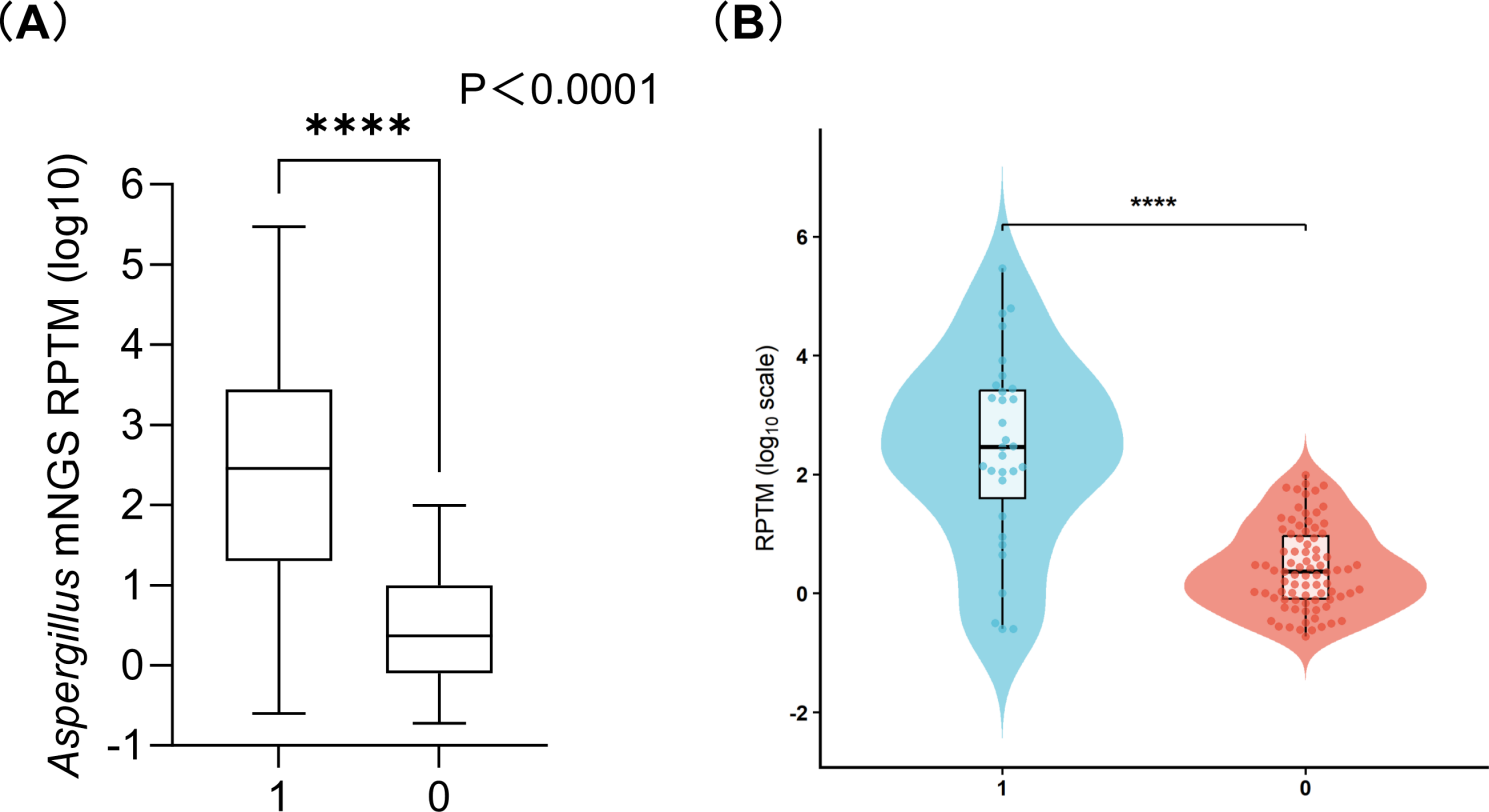
Distribution of *Aspergillus* RPTM in clinically diagnosed IPA and non-IPA groups. **(A)** Box plot and **(B)** violin plot showing log10-transformed RPTM values. Each point represents one patient. Boxes indicate the median and interquartile range. RPTM, reads per ten million. IPA, invasive pulmonary aspergillosis **** indicates P < 0.0001.

Among *Aspergillus*-positive patients, 31 met clinical diagnostic criteria for IPA, and the others were classified as non-clinically diagnosed IPA. Baseline characteristics, laboratory indices (WBC, NLR, CRP, PCT, D-dimer, TP, Alb), concurrent infections, dyspnea and hypoxemia were similar between the two groups (all P > 0.05, **Table 2**). *Aspergillus* RPTM was significantly higher in clinically diagnosed IPA than in non-clinically diagnosed IPA patients on the logarithmic scale (2.46 (IQR,1.16,3.47) vs.0.30 (IQR, -0.14,0.71); P < 0.001, **Figures 2A, B**), as were BALF galactomannan (GM) levels (0.15 (IQR, 0.00,3.55) vs. 0.00 (IQR, 0.00,0.00); P < 0.001). Antifungal therapy was also more frequently applied in patients with clinically diagnosed IPA (74.2% vs. 14.5%; P < 0.001).

**Table 2:** Baseline characteristics of patients with and without clinically diagnosed IPA.

Characteristics	Clinically diagnosed IPA (N = 31)	Non-clinically diagnosed IPA (N = 83)	P
Age (years)	69 (59,76)	65 (55,72)	0.352
BMI	24.18 ± 3.70	23.77 ± 4.22	0.232
Gender			0.894
Male	15 (48.4)	39 (47.0)	
Female	16 (51.6)	44 (53.0)	
Underlying diseases and immune status, n (%)			
COPD	9 (29.0)	18 (21.7)	0.412
Asthma	4 (12.9)	10 (12.0)	1.000
Bronchiectasis	10 (32.3)	14 (16.9)	0.073
Pulmonary underlying disease	19 (61.3)	34 (41.0)	0.053
Immunosuppression	15 (48.4)	40 (48.2)	0.985
Autoimmune disease/long-term steroid/immunosuppressant	6 (19.4)	13 (15.7)	0.638
Diabetes mellitus	3 (9.7)	19 (22.9)	0.112
Clinical laboratory tests, median			
WBC	6.58 (5.06,9.86)	6.47 (4.90,8.96)	0.408
Lymphocyte count	1.22 (0.88,1.39)	1.27 (0.86,1.82)	0.118
Neutrophil count	4.45 (3.00,7.61)	4.34 (3.05,6.97)	0.271
NLR	3.40 (2.35,8.42)	3.01 (1.96,6.86)	0.116
CRP (mg/L)	5.73 (1.89,40.16)	5.58 (1.26,28.79)	0.279
PCT(ng/mL)	0.042(0.025,0.083)	0.032 (0.021,0.067)	0.163
D-Dimer(ng/ml)	200 (111,499)	163 (104,380)	0.598
TP	68.40(63.65,73.25)	69.20 (65.25,73.75)	0.653
Alb	38.90(35.10,41.85)	39.70 (36.10,42.10)	0.777
Etiological detection indicators			
RPTM	286.07(14.49,2971.54)	2.00 (0.73,5.07)	<0.001*
BALF GM	0.15 (0.00,3.55)	0.00 (0.00,0.00)	<0.001*
Bacterial infection	15 (48.4)	48 (57.8)	0.367
Viral infection	4 (12.9)	7 (8.4)	0.487
Combined infection	17 (54.8)	50 (60.2)	0.602
Symptoms, n (%)			
Dyspnea	20 (64.5)	44 (53.0)	0.271
Hypoxemia	12 (38.7)	34 (42.0)	0.753
Treatment, n (%)			
Glucocorticoid use	10 (32.3)	31 (37.3)	0.614
Antifungal therapy	23 (74.2)	12 (14.5)	<0.001*

RPTM values are presented on the raw scale; logarithmic-scale values are reported in the text (**Figures 2A, B**).

NLR,Neutrophil-to-lymphocyte ratio; TP,total protein; Alb,albumin; BALF, bronchoalveolar lavage fluid; GM, galactomannan. *Indicates statistical significance at P < 0.05.

**Figure 3:**
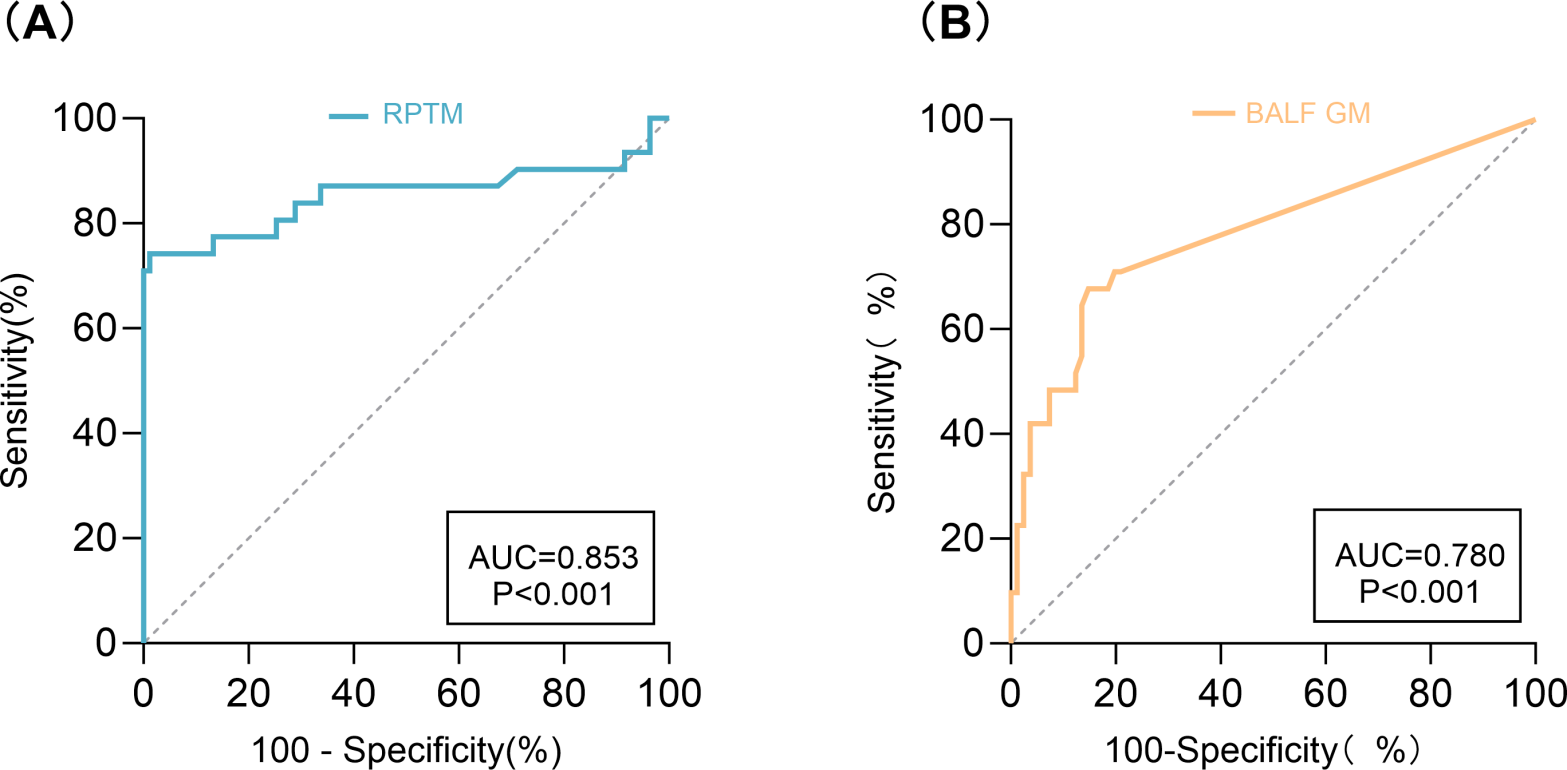
**(A)** Receiver operating characteristic (ROC) curve analysis for determining the optimal RPTM cut-off value to distinguish between the two groups. **(B)** ROC curve analysis for determining the optimal BALF GM cut-off value to distinguish between the two groups. mNGS, metagenomic next-generation sequencing; RPTM, reads per ten million; BALF, bronchoalveolar lavage fluid; GM, galactomannan.

ROC analysis demonstrated good discrimination for clinical IPA diagnosis, with an AUC of 0.853 (95% CI, 0.745-0.960; P < 0.001, **Figure 3A**). The exploratory cohort-derived RPTM threshold determined by the Youden index was 75. For comparison, BALF GM showed an AUC of 0.780 (95% CI, 0.674–0.886; P < 0.001, **Figure 3B**). Serum GM was not included in ROC analysis because quantitative serum GM ODI values were not consistently available.

This exploratory threshold provides a quantitative reference for interpreting *Aspergillus*-positive BALF mNGS results, but should be considered hypothesis-generating rather than clinically definitive. RPTM values above this level are more consistent with clinically diagnosed invasive disease, whereas lower values are more likely to represent colonization or non-invasive detection when interpreted in the appropriate clinical context. External validation is needed before this threshold can be applied clinically.

### 3.4 Higher fungal burden was associated with respiratory deterioration in untreated patients

Among 77 patients who did not receive antifungal therapy, 31 developed respiratory deterioration during 3-month follow-up and 46 did not. Baseline demographic characteristics, chronic pulmonary diseases, immunosuppression, diabetes mellitus, and most routine laboratory indices were similar between the groups.

Untreated patients who developed deterioration had significantly higher CRP levels than those without deterioration (16.38(IQR,3.78,54.41) vs. 3.40 (IQR, 1.01,14.20) mg/L; P = 0.010) and higher D-dimer levels (316 (IQR, 160,591) vs. 150(IQR, 101,347) ng/mL; P = 0.011). *Aspergillus* RPTM was also higher in the deterioration group (3.28 (IQR, 1.03,15.17) vs. 1.40 (IQR, 0.52,3.27); P = 0.007). BALF GM, bacterial infection, viral infection, combined infection, dyspnea, hypoxemia, and glucocorticoid use did not differ significantly between groups (**Table 3**).

**Table 3:** Baseline characteristics of untreated patients with and without respiratory deterioration.

Characteristics	RD (N = 31)	Non-RD (N = 46)	P
Age (years)	69 (58,74)	65 (59,70)	0.222
BMI	25.08 ± 4.07	23.46 ± 4.22	0.362
Gender			0.677
Male	17 (54.8)	23 (50.0)	
Female	14 (45.2)	23 (50.0)	
Underlying diseases and immune status, n (%)			
COPD	7 (22.6)	9 (19.6)	0.749
Asthma	3 (9.7)	4 (8.7)	1.000
Bronchiectasis	5 (16.1)	9 (19.6)	0.701
Pulmonary underlying disease	11 (35.5)	18 (39.1)	0.746
Immunosuppression	18 (58.1)	24 (52.2)	0.611
Autoimmune disease/long-term steroid/immunosuppressant	6 (19.4)	8 (17.4)	0.827
Diabetes mellitus	7 (22.6)	10 (21.7)	0.930
Clinical laboratory tests, median			
WBC	7.01(5.37,11.12)	6.36(4.94,8.67)	0.467
Lymphocyte count	1.11(0.86,1.72)	1.27(0.82,1.93)	0.400
Neutrophil count	4.87(3.46,8.49)	4.03(2.83,6.68)	0.269
NLR	5.00(2.38,7.88)	2.72(1.95,4.84)	0.131
CRP (mg/L)	16.38(3.78,54.41)	3.40(1.01,14.20)	0.010*
PCT (ng/mL)	0.047(0.019,0.071)	0.031(0.023,0.048)	0.158
D-Dimer (ng/ml)	316(160,591)	150(101,347)	0.011*
TP	69.65 ± 5.86	69.14 ± 5.43	0.644
Alb	38.10(32.23,42.80)	39.70(37.20,41.80)	0.191
Etiological detection indicators			
RPTM	3.28(1.03,15.17)	1.40(0.52,3.27)	0.007*
BALF GM	0.00(0.00,0.12)	0.00(0.00,0.00)	0.832
Bacterial infection	19 (61.3)	25 (54.3)	0.546
Viral infection	3 (9.7)	4 (8.7)	1.000
Combined infection	21 (67.7)	25 (54.3)	0.240
Symptoms, n (%)			
Dyspnea	16 (51.6)	23 (50.0)	0.890
Hypoxemia	11 (36.7)	23 (51.1)	0.218
Treatment, n (%)			
Glucocorticoid use	15 (48.4)	18 (39.1)	0.421

RD, respiratory deterioration; *Indicates statistical significance at P < 0.05.

### 3.5 Quantitative fungal burden showed modest discriminatory ability for respiratory deterioration

**Figure 4:**
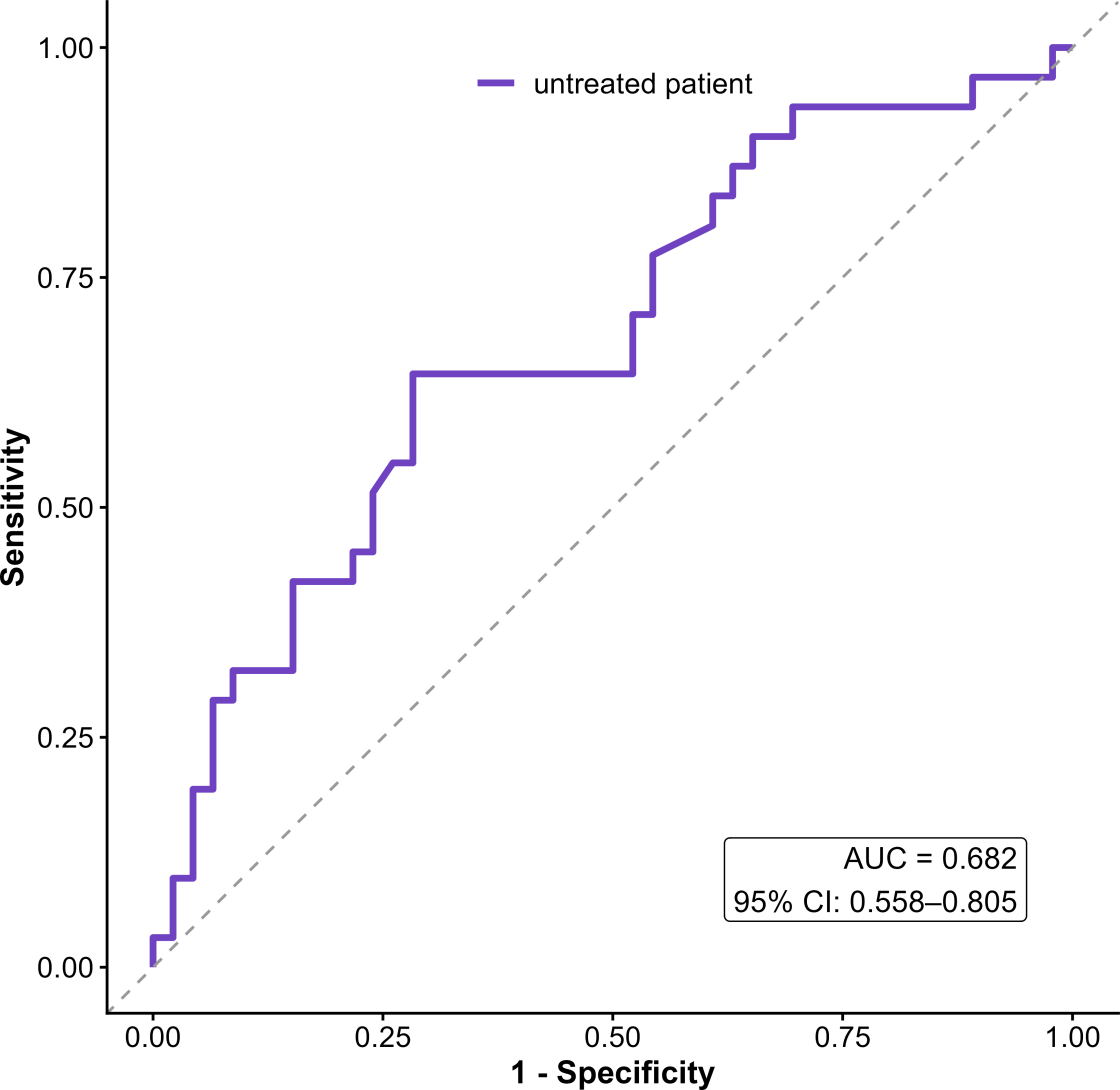
ROC curve analysis of mNGS RPTM for predicting respiratory deterioration risk in untreated patients.

ROC analysis showed that *Aspergillus* RPTM had modest discriminatory ability for subsequent respiratory deterioration in untreated patients, with an AUC of 0.682 (95% CI, 0.558–0.805; P = 0.0071, **Figure 4**). An exploratory cohort-derived RPTM threshold of 2.5 was identified by the Youden index and was used for subsequent exploratory regression analysis.

In univariable analysis, RPTM above 2.5 was associated with deterioration (OR, 4.81; 95% CI, 1.70-14.57; P = 0.004), as was CRP (OR, 1.01; 95% CI, 1.00-1.02; P = 0.040). D-dimer was not statistically significant in regression modeling. In multivariable analysis, elevated RPTM remained independently associated with deterioration after adjustment for CRP and D-dimer (adjusted OR, 5.27; 95% CI, 1.78-17.06; P = 0.001, **Table 4**).

**Table 4:** Factors associated with respiratory deterioration in untreated patients.

Factor	Univariate analysis		Multivariate analysis	
	OR (95% CI)	P	Adjusted OR (95% CI)	P
CRP	1.01 (1.00–1.02)	0.040	1.01 (1.00–1.03)	0.040*
D-dimer	1.00 (1.00–1.00)	0.580	1.00 (1.00–1.00)	0.926
RPTM > 2.5	4.81(1.70–14.57)	0.004*	5.27 (1.78–17.06)	0.001*

OR, odds ratio; 95% CI, 95% confidence interval; CRP, C-reactive protein; RPTM > 2.5 was defined as a binary variable based on the exploratory cohort-derived RPTM threshold of 2.5 derived from ROC analysis. Values > 2.5 were coded as 1, and values ≤ 2.5 were coded as 0. *Indicates statistical significance at P < 0.05.

### 3.6 Quantitative fungal burden improves clinical risk stratification

**Figure 5:**
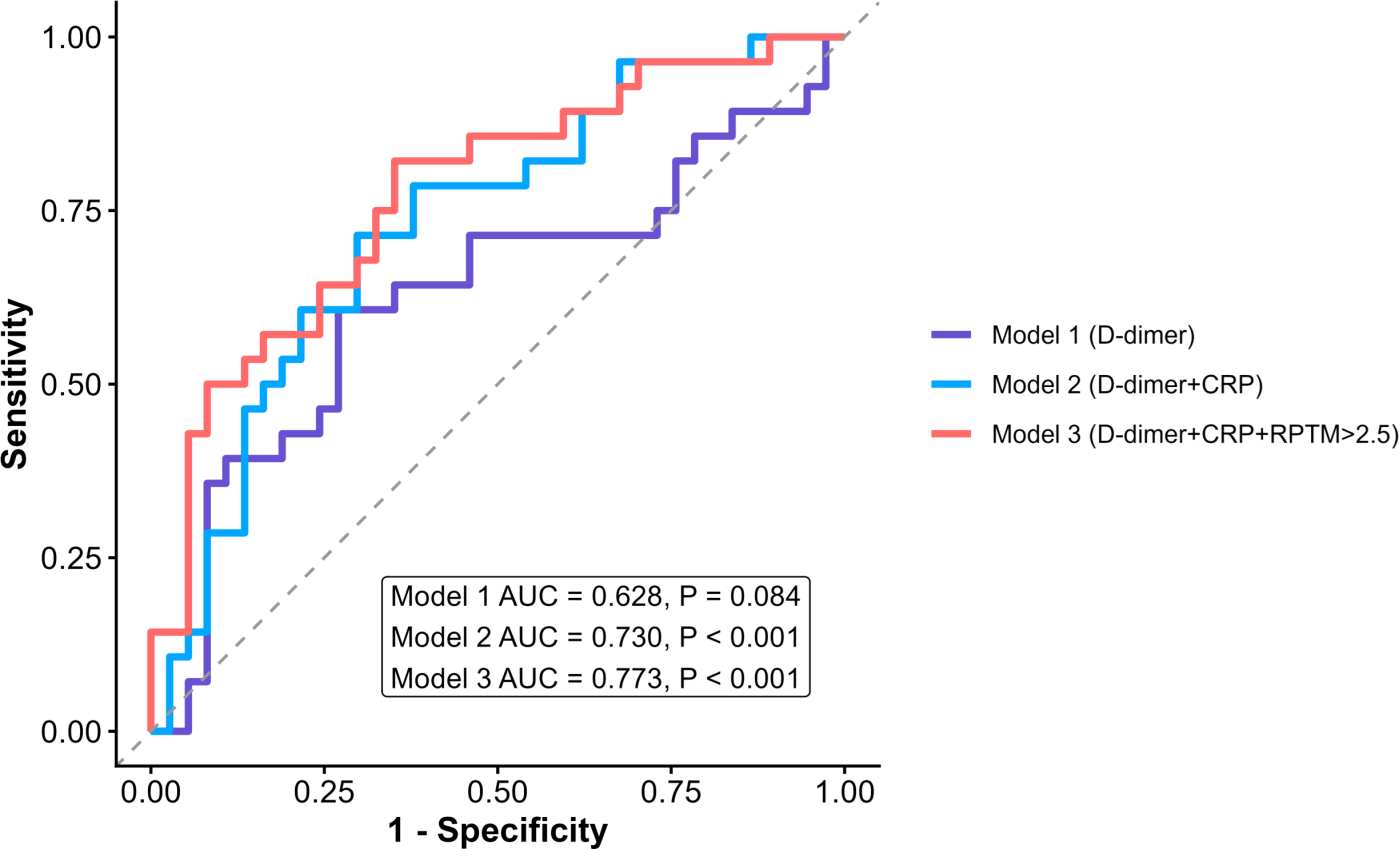
ROC curves of sequential models for predicting respiratory deterioration in untreated patients. Model 1: D-dimer. Model 2: D-dimer + CRP. Model 3: D-dimer + CRP + RPTM (>2.5). CRP, C-reactive protein; RPTM, reads per ten million.

To assess whether quantitative *Aspergillus* burden added prognostic information beyond conventional clinical variables, sequential prediction models were constructed in untreated patients (**Table 5**). Model 1 included D-dimer and yielded an AUC of 0.628 (95% CI, 0.483-0.773; P = 0.084). Addition of CRP generated Model 2 and improved the AUC to 0.730 (95% CI, 0.605-0.854; P<0.001). Further addition of NGS binary based on RPTM >2.5 generated Model 3 and improved the AUC to 0.773 (95% CI, 0.657-0.890; P<0.001, **Figure 5**).

**Table 5:** Discriminatory performance of sequential models for predicting respiratory deterioration in untreated patients.

Model	Original AUC	Original 95% CI	Corrected AUC	SE	Corrected 95% CI
Model 1 (D-Dimer)	0.628	0.483-0.773	0.627	0.075	0.495–0.779
Model 2 (D-Dimer + CRP)	0.730	0.605-0.854	0.748	0.071	0.571–0.835
Model 3 (Full)	0.773	0.657-0.890	0.762	0.058	0.659–0.882

To reduce overfitting and optimism bias, bootstrap internal validation (200 resamples) was performed. The optimism-corrected AUC was 0.627 (95% CI, 0.495–0.779) for Model 1, 0.748 (95% CI, 0.571–0.835) for Model 2, and 0.762 (95% CI, 0.659–0.882) for Model 3.

IDI and category-free NRI analyses supported the incremental value of quantitative mNGS burden (**Table 6**). Addition of CRP to Model 1 improved IDI by 0.074 (95% CI, 0.012-0.136; P = 0.020) and category-free NRI by 0.604 (95% CI, 0.218-0.990; P<0.001). Further addition of NGS binary improved IDI by 0.126 (95% CI, 0.049-0.204; P<0.001) and category-free NRI by 0.728 (95% CI, 0.295-1.166; P<0.001).

**Table 6:** Incremental predictive value of CRP and quantitative *Aspergillus* burden for respiratory deterioration in untreated patients.

Model comparison	IDI			Category-free NRI		
	Index	95% CI	P	Index	95% CI	P
Model1/Model1+CRP (Model2)	0.074	0.012–0.136	0.020	0.604	0.218–0.990	<0.001
Model2/Model2+RPTM>2.5 (Model3)	0.126	0.049-0.204	<0.001	0.728	0.295-1.166	<0.001

### 3.7 Baseline fungal burden was not associated with respiratory deterioration in antifungal-treated patients

Among 37 patients who received antifungal therapy, 9 developed respiratory deterioration and 28 did not. Baseline demographic characteristics, underlying diseases, immune status, routine laboratory indices, and microbiological variables were generally similar between the two groups (**Table 7**). Importantly, baseline *Aspergillus* RPTM did not differ significantly between treated patients with and without subsequent deterioration (116.00(IQR,3.97,333.04) vs.123.68(IQR, 16.61,2693.85); P = 0.519). Because RPTM was not associated with deterioration in this subgroup, no further ROC analysis was performed.

**Table 7:** Baseline characteristics of treated patients with and without respiratory deterioration.

Characteristics	RD (N =9)	Non-RD (N = 28)	P
Age (years)	68 (57,78)	66 (52,76)	0.848
BMI	23.4(21.7,23.8)	22.3(20,25.3)	0.647
Gender			0.705
Male	4 (44.4)	10 (35.7)	
Female	5 (55.6)	18 (64.3)	
Underlying diseases and immune status, n (%)			
COPD	2 (22.2)	9 (32.1)	0.695
Asthma	1 (11.1)	6 (21.4)	0.656
Bronchiectasis	2 (22.2)	8 (28.6)	1.000
Pulmonary underlying disease	4 (44.4)	20 (71.4)	0.229
Immunosuppression	2 (22.2)	11 (39.3)	0.446
Autoimmune disease/long-term steroid/immunosuppressant	1 (11.1)	4 (14.3)	1.000
Diabetes mellitus	1 (11.1)	4(14.3)	1.000
Clinical laboratory tests, median			
WBC	8.09(4.21,9.13)	5.96 (5.02,8.29)	0.667
Lymphocyte count	1.31(0.95,1.45)	1.28 (0.94,1.64)	0.903
Neutrophil count	6.47(2.98,6.98)	3.94 (3.11,6.20)	0.715
NLR	4.10(2.25,5.70)	2.87 (2.00,7.21)	0.903
CRP (mg/L)	18.03(1.92,66.55)	3.08(0.94,16.94)	0.239
PCT (ng/mL)	0.041(0.035,0.117)	0.039(0.020,0.068)	0.086
D-Dimer (ng/ml)	197(112,435)	149 (103,295)	0.903
TP	70.29 ± 9.23	68.36 ± 7.57	0.619
Alb	39.08 ± 3.57	39.50 ± 5.61	0.958
Etiological detection indicators			
RPTM	116.00(3.97,333.04)	123.68(16.61,2693.85)	0.519
BALF GM	0.00(0.00,0.08)	0.13 (0.00,1.85)	0.056
Bacterial infection	3 (33.3)	16 (57.1)	0.269
Viral infection	1 (11.1)	3 (10.7)	1.000
Combined infection	3 (33.3)	18 (64.3)	0.136
Symptoms, n (%)			
Dyspnea	4 (44.4)	21 (75.0)	0.116
Hypoxemia	4 (44.4)	8 (28.6)	0.432
Treatment, n (%)			
Glucocorticoid use	1 (11.1)	7 (25.0)	0.649

## 4 Discussion

The widespread implementation of bronchoalveolar lavage fluid (BALF) metagenomic next-generation sequencing (mNGS) has fundamentally changed the diagnostic landscape of pulmonary fungal diseases (Ji et al., 2026; Yang et al., 2021). While mNGS has substantially improved the sensitivity of *Aspergillus* detection, interpretation of *Aspergillus*-positive results remains a major challenge in clinical practice (Sun et al., 2024). The key dilemma is that *Aspergillus* detection does not necessarily indicate invasive pulmonary aspergillosis (IPA), particularly in patients with chronic respiratory diseases, structural lung abnormalities, or non-classical host factors, in whom airway colonization is common (Pham et al., 2025). Consequently, clinicians are increasingly confronted with the question of how to distinguish clinically significant infection from colonization and determine which patients may benefit from antifungal therapy. In this real-world cohort of 114 patients with *Aspergillus*-positive BALF mNGS results, we demonstrated that quantitative fungal burden provides clinically meaningful information beyond qualitative pathogen detection alone. According to the EORTC/MSGERC criteria, all 31 clinically diagnosed IPA patients in this cohort met the definition of probable IPA, reflecting the diagnostic reality that histopathological confirmation—required for proven IPA—is rarely obtainable in real-world non-neutropenic populations. The additional identification of 2 possible IPA cases within the non-IPA group further underscores the diagnostic gray zone in patients with host factors and compatible imaging but without sufficient mycological evidence.

The principal findings of this study are fourfold. First, *Aspergillus* burden measured by reads per ten million (RPTM) was markedly higher in patients with clinically diagnosed IPA than in non-IPA patients, and an exploratory RPTM threshold of 75 showed good discrimination for clinical IPA adjudication. Second, among patients who did not receive antifungal therapy, elevated *Aspergillus* burden was associated with subsequent respiratory deterioration during follow-up. Third, quantitative fungal burden remained independently associated with deterioration after adjustment for conventional inflammatory markers and improved risk discrimination and reclassification in exploratory models. Finally, in the antifungal-treated subgroup, baseline RPTM was not significantly associated with subsequent respiratory deterioration. This finding should be interpreted cautiously because of the small sample size, non-randomized treatment allocation, and potential confounding by indication.

One of the most clinically relevant findings of the present study is the strong association between quantitative *Aspergillus* burden and clinical IPA diagnosis. Although mNGS has demonstrated superior sensitivity compared with conventional microbiological methods, the increased detection rate of *Aspergillus* has simultaneously created a diagnostic challenge because qualitative positivity alone lacks specificity for invasive disease. Previous studies have reported that *Aspergillus* read counts derived from BALF mNGS may correlate with IPA diagnosis; however, most investigations have focused primarily on diagnostic accuracy and included relatively selected populations (Zhan et al., 2023; Jia et al., 2023; Liu et al., 2026). Our findings are consistent with these observations and further demonstrate that fungal burden differs dramatically between clinically diagnosed IPA and non-IPA patients in a heterogeneous real-world cohort that included non-neutropenic individuals, patients with chronic respiratory diseases, and those with metabolic comorbidities. The approximately 100-fold difference in median RPTM between IPA and non-IPA groups suggests that quantitative sequencing burden captures biologically relevant information beyond simple organism detection.

The exploratory diagnostic threshold identified in this study provides a reference for future research and clinical hypothesis generation. Rather than treating *Aspergillus* positivity as a binary variable, clinicians may benefit from considering the magnitude of fungal burden when interpreting mNGS results. Patients with substantially elevated RPTM values are more likely to have active fungal proliferation and invasive disease, whereas very low fungal burdens may reflect colonization, transient environmental exposure, or clinically insignificant detection (Zhou et al., 2025). Such an approach is particularly important in non-neutropenic patients, in whom classical radiological manifestations and host factors often fail to provide sufficient diagnostic certainty (Wang et al., 2025). Notably, typical imaging findings associated with IPA, including halo sign and cavitary lesions, were uncommon in our cohort, whereas nonspecific abnormalities such as bilateral infiltrates, nodules, patchy opacities, and ground-glass changes predominated. This observation further highlights the need for adjunctive biomarkers capable of improving diagnostic stratification.

Beyond diagnostic discrimination, our study extends the potential utility of quantitative *Aspergillus* burden into the prognostic domain. To our knowledge, few previous studies have specifically examined whether quantitative fungal burden is associated with future clinical outcomes among *Aspergillus*-positive patients who do not initially receive antifungal therapy. We observed that untreated patients who subsequently developed respiratory deterioration had significantly higher baseline RPTM values than those who remained clinically stable. Furthermore, elevated fungal burden remained independently associated with deterioration after adjustment for CRP and D-dimer levels, suggesting that the association was not merely a reflection of systemic inflammation or disease severity. Given the modest AUC, RPTM should be interpreted as an adjunctive risk-stratification marker rather than a standalone predictor of respiratory deterioration.

Several biological mechanisms may explain this observation. First, elevated *Aspergillus* burden may indicate active fungal proliferation within the respiratory tract rather than passive colonization (Shinfuku et al., 2023). Progressive fungal growth can promote persistent airway inflammation, epithelial injury, and disruption of mucosal barrier integrity, thereby increasing susceptibility to secondary infections and respiratory deterioration (Wang et al., 2026). Second, *Aspergillus*-derived proteases, toxins, and pathogen-associated molecular patterns can activate innate immune pathways and amplify local inflammatory responses. Excessive inflammatory activation may contribute to progressive pulmonary injury even in the absence of classical invasive disease (Shankar et al., 2024). Third, high fungal burden may serve as a surrogate marker of impaired local host defense mechanisms, reflecting an inability of the host immune system to adequately control fungal growth. These mechanisms are not mutually exclusive and may collectively contribute to the observed association between fungal burden and adverse clinical outcomes.

An additional strength of this study is the demonstration that quantitative fungal burden provides incremental prognostic information beyond conventional clinical markers. Although CRP and D-dimer are widely used indicators of inflammation and disease severity, neither is specific for fungal disease. By incorporating RPTM into predictive models, we observed significant improvements in discrimination and patient reclassification, as demonstrated by both IDI and category-free NRI analyses. These findings suggest that fungal burden reflects a distinct biological dimension that is not captured by routine laboratory variables. Consequently, quantitative mNGS interpretation may facilitate more accurate identification of patients at increased risk for deterioration and support individualized monitoring strategies.

Perhaps the most intriguing finding of the present study is the apparent disappearance of the association between fungal burden and respiratory deterioration among patients who received antifungal therapy. However, this finding must be interpreted with considerable caution. The sample size of this subgroup was small (only 37 patients, of whom 9 experienced deterioration), treatment allocation was not randomized, and there is a high likelihood of confounding by indication—patients with higher RPTM values, more abnormal radiological findings, or a stronger clinical suspicion of invasive pulmonary aspergillosis were more likely to receive antifungal therapy. As a result, the present study cannot reliably determine whether antifungal therapy attenuates the prognostic significance of fungal burden. However, any potential biological mechanism underlying this observation remains speculative and cannot be inferred from the present study due to its observational design and confounding by indication (Wang et al., 2025). Given the study’s limitations, clinical decisions should not be based on this subgroup finding, and further research is needed before clinical implications can be determined. If confirmed in larger, prospective, and appropriately designed studies, this observation might eventually help identify a subgroup of *Aspergillus*-positive patients who do not meet criteria for definite invasive pulmonary aspergillosis but may still benefit from earlier therapeutic intervention.

**Figure 6:**
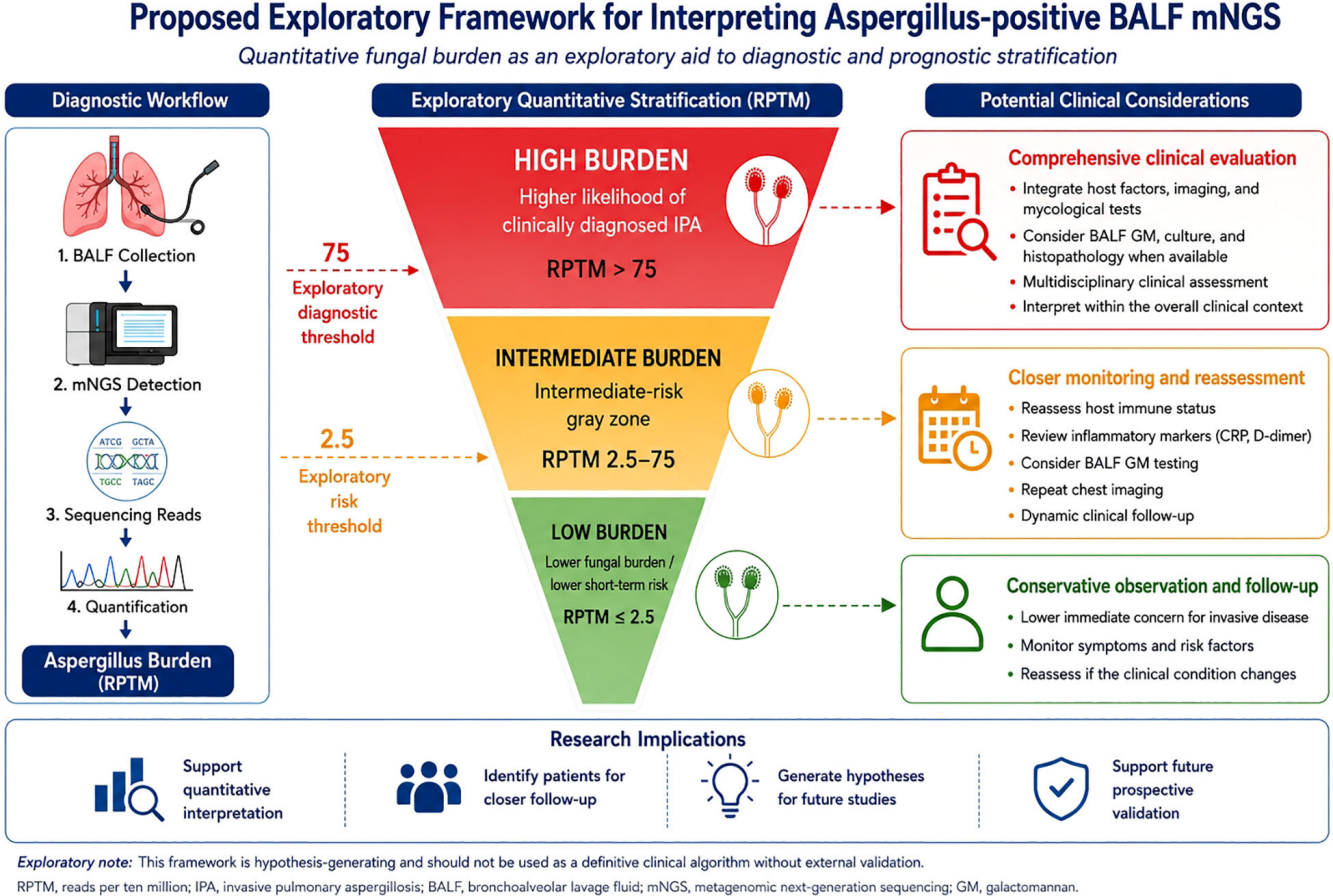
Proposed exploratory framework for interpreting *Aspergillus*-positive BALF mNGS. The framework illustrates how quantitative *Aspergillus* burden may support exploratory diagnostic and prognostic stratification across high-, intermediate-, and low-burden ranges. This framework is hypothesis-generating and should not be used as a definitive clinical algorithm without external validation. RPTM, reads per ten million; IPA, invasive pulmonary aspergillosis; BALF, bronchoalveolar lavage fluid; mNGS, metagenomic next-generation sequencing; GM, galactomannan.

Based on the exploratory diagnostic and prognostic thresholds identified in this study, we propose a preliminary quantitative, risk-stratified framework for interpreting *Aspergillus*-positive BALF mNGS results (**Figure 6**). This framework is intended to generate hypotheses for future prospective studies and should not be used as a definitive clinical algorithm without external validation. Rather than treating *Aspergillus* detection as a binary positive or negative finding, quantitative fungal burden may facilitate risk stratification across a continuum. Patients with markedly elevated fungal burdens exceeding the diagnostic threshold are more likely to have invasive disease and should undergo comprehensive evaluation for IPA. Conversely, patients with very low fungal burdens may be managed conservatively with routine follow-up, as clinically significant infection appears less likely. Between these extremes lies an intermediate-risk zone, in which fungal burden alone may be insufficient to establish a diagnosis of IPA but may nevertheless identify patients who require closer reassessment. In such patients, serial imaging, inflammatory markers, galactomannan testing, and repeated clinical evaluation may help refine risk assessment. This exploratory framework moves mNGS interpretation beyond a simple positive-versus-negative result and provides a basis for future prospective validation rather than a definitive clinical algorithm.

Several limitations should be acknowledged. First, this was a retrospective single-center study with a relatively modest sample size, which may limit the generalizability of the findings and the precision of the threshold estimates. The RPTM thresholds and predictive models developed in this study should therefore be regarded as exploratory tools for risk stratification rather than definitive clinical prediction models for immediate clinical use. Although bootstrap internal validation was performed, the limited number of deterioration events may still have introduced model instability and overfitting.

Second, the follow-up duration was limited to 3 months, which restricts the evaluation of longer-term clinical outcomes. This relatively short observation period may have underestimated late respiratory deterioration, particularly in patients with chronic progressive pulmonary diseases. Future prospective studies with extended follow-up are warranted to determine whether the association between baseline fungal burden and clinical outcomes persists beyond the early post-discharge period.

Third, histopathological confirmation was unavailable in most patients, and IPA classification relied on multidisciplinary clinical assessment and established diagnostic criteria. Although this approach reflects real-world practice, diagnostic misclassification remains possible. Fourth, although BALF GM was available for ROC comparison, quantitative serum GM ODI values were not systematically available, which precluded a reliable ROC analysis for serum GM. In addition, species-level assignments were based on retrospective mNGS reports and were not systematically confirmed by culture-based sequencing or antifungal susceptibility testing; therefore, species-specific burden analyses, particularly for uncommon or potentially cryptic *Aspergillus* species, should be interpreted cautiously. Fifth, mNGS platforms differ in sequencing depth, bioinformatics pipelines, reference databases, and contamination-control strategies; therefore, the RPTM thresholds identified in this cohort should not be directly extrapolated to other laboratories without external validation. Finally, antifungal treatment was not randomly assigned and may have been influenced by physician judgment, disease severity, radiological findings, and perceived likelihood of invasive disease, introducing confounding by indication.

Future prospective multicenter studies are needed to validate the thresholds identified in this study and determine their applicability across different sequencing platforms and patient populations. In addition, integration of quantitative fungal burden with galactomannan, host immune biomarkers, radiomics, and machine-learning approaches may further improve diagnostic and prognostic performance. Serial mNGS monitoring may also provide valuable insights into treatment response and disease dynamics. Ultimately, development of standardized quantitative interpretation criteria will be essential for translating mNGS findings into routine clinical decision-making.

## 5 Conclusion

Our study demonstrates that quantitative *Aspergillus* burden derived from BALF mNGS provides clinically meaningful information beyond qualitative positivity alone. Higher fungal burden was strongly associated with clinically diagnosed IPA and was independently associated with respiratory deterioration among untreated patients. The absence of this association in antifungal-treated patients should be interpreted with caution due to small sample size and confounding by indication. These findings support a quantitative interpretation framework for *Aspergillus*-positive BALF mNGS results and may facilitate more precise diagnosis, risk stratification, and individualized management of pulmonary *Aspergillus* disease. However, RPTM should be interpreted as an adjunctive risk-stratification tool rather than a standalone predictive marker, and the findings require validation in larger prospective cohorts.

## Data Availability

The raw metagenomic sequencing data analyzed in this study were generated and retained by a third-party clinical laboratory as part of routine clinical testing. Under the laboratory’s data management policy, these data are not disclosed to external parties or deposited in public repositories without client authorization or a specific client request. The authors do not directly hold or independently control the raw sequencing files; therefore, no public repository accession number is currently available. De-identified clinical data and processed data supporting the findings of this study may be made available from the corresponding author upon reasonable request, subject to applicable ethical, institutional, and data-protection requirements. Requests for access to the raw metagenomic sequencing data may be considered through the corresponding author and would additionally require authorization and cooperation from the third-party laboratory.
